# Childhood cancer in the offspring born in 1921–1984 to US radiologic technologists

**DOI:** 10.1038/sj.bjc.6604516

**Published:** 2008-07-29

**Authors:** K J Johnson, B H Alexander, M M Doody, A J Sigurdson, M S Linet, L G Spector, R W Hoffbeck, S L Simon, R M Weinstock, J A Ross

**Affiliations:** 1 Department of Pediatrics, Division of Epidemiology/Clinical Research, University of Minnesota, Minneapolis, MN 55455, USA; 2 Division of Environmental Health Sciences, University of Minnesota, Minneapolis, MN 55455, USA; 3 Radiation Epidemiology Branch, Division of Cancer Epidemiology and Genetics, National Cancer Institute, National Institutes of Health, Bethesda, MD 20892, USA; 4 University of Minnesota Cancer Center, Minneapolis, MN 55455, USA; 5 RTI, Rockville, MD 20852, USA

**Keywords:** radiation, *in utero*, preconception, malignancy, aetiology, risk factors

## Abstract

We examined the risk of childhood cancer (<20 years) among 105 950 offspring born in 1921–1984 to US radiologic technologist (USRT) cohort members. Parental occupational *in utero* and preconception ionising radiation (IR) testis or ovary doses were estimated from work history data, badge dose data, and literature doses (the latter doses before 1960). Female and male RTs reported a total of 111 and 34 haematopoietic malignancies and 115 and 34 solid tumours, respectively, in their offspring. Hazard ratios (HRs) and 95% confidence intervals (CIs) were calculated using Cox proportional hazards regression. Leukaemia (*n*=63) and solid tumours (*n*=115) in offspring were not associated with maternal *in utero* or preconception radiation exposure. Risks for lymphoma (*n*=44) in those with estimated doses of <0.2, 0.2–1.0, and >1.0 mGy *vs* no exposure were non-significantly elevated with HRs of 2.3, 1.8, and 2.7. Paternal preconception exposure to estimated cumulative doses above the 95th percentile (⩾82 mGy, *n*=6 cases) was associated with a non-significant risk of childhood cancer of 1.8 (95% CI 0.7–4.6). In conclusion, we found no convincing evidence of an increased risk of childhood cancer in the offspring of RTs in association with parental occupational radiation exposure.

Exposure to *in utero* diagnostic ionising radiation (IR) is generally considered to increase the risk of childhood cancer, including solid and haematological malignancies (Brenner *et al*, 2003; Wakeford, 2004). Although cohort studies of medical radiation workers have reported increases in the incidence and mortality of skin cancer, breast cancer, and leukaemia, especially in individuals who started working before 1950 when both radiation doses and permitted levels of exposure were higher (Yoshinaga *et al*, 2004), the risk of cancer in their offspring associated with parental occupational exposure is unclear (Brenner *et al*, 2003). Radiation could increase offspring cancer risk through germline or *in utero* somatic mutations (Doll and Wakeford, 1997; UNSCEAR, 2001; Prasad *et al*, 2004). The only study of childhood cancer incidence in the offspring of medical radiographers found no significant excess (Roman *et al*, 1996).

The US radiologic technologist (USRT) cohort is one of the largest groups of occupationally exposed medical personnel assembled for study. As 73% USRT cohort participants are female, both preconception and *in utero* occupational radiation exposure effects on offspring cancer risk could be studied. In addition, much effort was devoted to reconstructing probable dose levels during periods when workers were not routinely monitored (Simon *et al*, 2006), allowing quantitative evaluation of parental exposures. To date, this is the largest study of childhood cancer risk in RT offspring.

## Methods

The University of Minnesota Institutional Review Board and the United States National Cancer Institute approved all protocols for data use. Complete study details have been described elsewhere (Boice *et al*, 1992; Doody *et al*, 1998; Mohan *et al*, 2002, 2003; Freedman *et al*, 2003; Sigurdson *et al*, 2003). Briefly, three questionnaires were mailed to individuals registered with the American Registry of Radiologic Technologists during 1983–89, 1994–98, and 2004–08. The initial questionnaire was sent to 132 454 RTs and returned by 90 305 of them, a response rate of 68%, whereas 83% of RTs (70 859 out of 85 372) who responded to the first questionnaire returned the second one of which 132 118 offspring were enumerated. The following information was reported on the second questionnaire for up to eight of their children: gender; birth year; the occurrence of cancer (no, yes, do not know) and its type (leukaemia, lymphoma, brain, or ‘other, specify’; the latter detail was entered into the data file verbatim); diagnosis year; vital status; and death year. We excluded offspring who were born after 1984 (*n*=23 123; the latest year for which estimated doses were available); had missing, incomplete, or inconsistent data on birth year, parent birth year, cancer diagnosis year, or death year (*n*=2859); had reported Down's syndrome (*n*=123); or had an ‘other cancer’ reported as a condition of the skin (other than sarcoma) or the cervix (*n*=73) occurring during childhood (<20 years), to eliminate potentially benign common conditions of these tissues (e.g., moles and abnormal pap smears). The resultant data set included 81 262 and 24 678 offspring of female and male RTs, respectively.

Annual occupational radiation doses received by RTs were estimated as described previously (Simon *et al*, 2006), using all relevant literature on doses received by RTs (mainly for those working before 1960, when dosimetry was not available), film-badge measurements from a commercial dosimetry provider or military dose registries, dose records provided by employers, and individual work history and protection practices from three cohort surveys. Annual ovary and testes organ dose estimates were used as the best approximations for foetal and germline exposures. The estimated *in utero* exposure dose was defined as the average of the ovarian doses in the year before and the year of birth, as only the year of birth rather than the date of birth was collected. The estimated preconception dose was calculated as the sum of annual testes or ovary dose estimates up to the year before birth to avoid overlap with the *in utero* period.

### Statistical analyses

Hazard ratios (HRs) and 95% confidence intervals (CIs) were calculated by Cox proportional hazards regression (SAS version 9.1; Cary, NC, USA). Person-time was calculated as the interval between the reported birth year and the cancer diagnosis year, death year, the year the child turned age 19, or the year that the second questionnaire was completed, whichever came first. A person-time of 0.5 years was assigned to those who were censored or had an event in their birth year. All regression analyses were stratified by the gender of the parent.

Average cancer incidence rates per million person-years were determined for each age group in years (0–4, 5–9, 10–14, 15–19) as the number of events per person-years. The ratio of observed to expected numbers (the standardised incidence ratio (SIR)) was calculated for RT parent and child gender strata for childhood leukaemia, lymphoma, and all sites by applying the sex-specific incidence rates obtained from two registries: (1) Surveillance, Epidemiology, and End Results (SEER) 9 (1973–2004) (SEER, 2007) and (2) Connecticut historical registry (rates available from 1935 to 1999) (SEER, 1999) to the person-time distribution of the offspring cohort (Rothman and Greenland, 1998). For the SEER registry comparison, rates for person-time for years before 1973 were compared to SEER incidence rates for 1975–76, the earliest period with complete data (SEER, 2007). Only subjects whose parents were reported to be white were ncluded due to the small number of other races.

Exposures were modelled as categorical variables with *P*-values for tests for linear trend calculated by inclusion of the continuous measure of dose in regression models. Estimated in utero dose exposures were categorized as follows: 0 mGy (reference), >0–0.17 mGy, 0.18–1.0 mGy, and 0.01–12.6 mGy. Estimated preconception ovarian and testes dose exposures were categorized according to quartiles: <0.43 mGy, 0.43–1.49 mGy, 1.50–3.57 mGy, >3.57 mGy; testes: <0.67, 0.67–4.92, 4.93–15.26, >15.27) with additional analyses for the offspring of males that divided the fourth quartile at the 95th percentile (>81.92 mGy).

For the offspring of female RTs, results are reported for the following childhood cancer outcomes: (1) leukaemia, (2) lymphoma, (3) solid tumours, and (5) all childhood cancers. Owing to the small number of childhood cancer cases reported by male RTs (*n*=68), results are given for haematological malignancies (leukaemia and lymphoma), solid tumours, and overall childhood cancers only.

## Results

Characteristics of all RTs and those with eligible children who responded to both the baseline and second questionnaires are provided in Supplementary Table 1. The majority of RT respondents were female (79%), born during 1941–1960 (78%), first employed during 1961–1980 (79%), and reported having had at least one child (80%) with a median of two children.

The characteristics of the offspring are presented in Table 1. The majority of offspring were born between 1960 and 1980 with slightly more male than female offspring, and a more pronounced male excess in cases of leukaemia (58% male) and lymphoma (72% male) (data not shown). The mean estimated *in utero* and preconception doses in the offspring of both female and male RTs generally decreased over time, declining approximately four- to sixfold from the 1930s through the 1970s and 1980s. Estimated doses were generally low and similar between cases and all subjects.

Approximately 96% of cases and 95% of non-cases had parents who reported being white. Cases and non-cases were similar with respect to birth order (48% of cases *vs* 46% of non-cases reported having a birth order of 1). Overall, case parents tended to be younger (<30 years) than non-case parents at the time of the offspring's birth (79 *vs* 73%) (data not shown).

In total, 294 offspring had reported cancers diagnosed at <20 years of age (Table 2). Leukaemia (*n*=94), lymphoma (*n*=61), and central nervous system (CNS) tumours (*n*=46) were the most common cancer types. The peak leukaemia incidence occurred at <5 years of age with rates of 72 and 97 cases per million person-years in female and male RT offspring, respectively. Peak lymphoma incidence occurred in older children, with the highest rate being in 15- to 19-year olds. Central nervous system tumour rates did not show marked variation by age in the offspring of female RTs. The pattern for the offspring of male RTs was less consistent, presumably due to the small number of cases (*n*=12).

No significant increase in risk or dose–response was found for leukaemia, lymphoma, solid tumours, or childhood cancer overall in association with *in utero* radiation exposure (Table 3). Based on 48 cases of lymphoma, the HR was increased approximately two- to threefold for all dose categories above the reference with no apparent linear trend (*P*=0.32).

No appreciable increased risk or dose–response was observed between maternal preconception exposure and childhood cancer. The estimated paternal doses above the 95th percentile (>82 mGy) were associated with a non-significant increased risk of 1.8 (95% CI 0.7–4.6) relative to the reference group, on the basis of six events (two leukaemia, two lymphoma, one sarcoma, and one oral cancer case) (Supplementary Table 2).

Standardised incidence ratios were calculated for leukaemia, lymphoma, or all reported childhood cancer rates in the offspring of RTs relative to SEER and Connecticut registry rates (Table 4). Cancer incidence was not increased in RT offspring for all sites combined or leukaemia. The incidence of lymphoma was increased in the male offspring of both female and male RTs relative to both population registries with SIRs between 1.6 and 1.7.

## Discussion

Overall, our results do not support an increased risk of childhood cancer in the offspring of RTs associated with occupational IR exposure while *in utero* or before conception. An increased risk of lymphoma was observed based on a small number of cases but there was no dose–response. Our data do not indicate an association between maternal preconception radiation exposure and offspring childhood cancers. A non-significant increased risk of childhood cancer was associated with paternal preconception exposure only above the 95th percentile (>82 mGy), based on few cases.

*In utero* exposure to maternal occupational medical radiation did not markedly increase the risk of any of the childhood cancer outcomes examined except lymphoma. A small increase of approximately 40% in the risk of childhood cancer is thought to be detectable for acute exposure to diagnostic *in utero* radiation at doses in the range of 10 mGy, based on results from case–control studies of subjects born between late 1940s and early 1980s (Doll and Wakeford, 1997). The offspring of RTs in our study were generally exposed to lower doses, with the highest estimated dose for cases being 3.3 mGy, for a subject with a reported brain tumour. The estimated mean *in utero* dose for lymphoma cases was 0.3 mGy, which is approximately 10-fold lower than average annual background radiation exposure (Wakeford, 2004) and, therefore, it is unlikely that maternal occupational radiation exposure explains the increased lymphoma risk.

Consistent with our results, most previous studies have not provided strong support for an association between childhood cancer and parental preconception exposures to either low or high doses of atomic bomb radiation (Izumi *et al*, 2003a, 2003b), medical or nuclear occupational radiation (Kinlen *et al*, 1993; McLaughlin *et al*, 1993; Roman *et al*, 1993, 1996; Draper *et al*, 1997; Pobel and Viel, 1997), or therapeutic or diagnostic medical radiation (Kallen *et al*, 1998; Sankila *et al*, 1998; Little, 1999; Shu *et al*, 2002; Patton *et al*, 2004; Nagarajan and Robison, 2005). A notable exception (Gardner *et al*, 1990) examined the incidence of leukaemia/non-Hodgkin's lymphoma (LNHL) diagnosed at an age of <25 years in individuals living near the Sellafield nuclear facility in England, which included 74 cases of LNHL (14 in the offspring of fathers employed at Sellafield). A significant increased risk of 6.4 for LNHL was reported in association with paternal cumulative preconception radiation dose >100 mSV on the basis of 4 exposed cases. The case excess was largely confined to the neighbouring village of Seascale (Cumbria, England). Subsequent independent investigations conducted in England, France, Scotland, and Canada have failed to support this association (Kinlen *et al*, 1993; McLaughlin *et al*, 1993; Draper *et al*, 1997; Pobel and Viel, 1997). The alternative hypothesis for Gardner's findings of population mixing (Kinlen, 1988) has been supported by several studies (Little, 1999; McNally and Eden, 2004).

Excess in cases of lymphoma in the male offspring of those exposed to radiation has not been previously reported. No male excess has been reported in association with *in utero* diagnostic radiation exposure in two of the largest studies: the Oxford Survey of Childhood Cancers (Bithell and Stewart, 1975) and the North-eastern United States study (Monson and MacMahon, 1984), or in the offspring of atomic bomb survivors who were exposed while *in utero* (Delongchamp *et al*, 1997).

Our study addresses the important issue of cancer risk in the offspring of medical radiation workers, particularly females, who are exposed to low-level protracted occupational radiation. Prior data comes mainly from studies of cancer risk in offspring of nuclear workers who are predominantly male. However, as with all studies of rare diseases and low-dose exposures, our study has its own limitations. Electronic files of film-badge doses were not routinely available until late 1970s. To estimate these doses, we undertook a comprehensive dose reconstruction that used hundreds of thousands of badge doses from electronic files from 1977 onward, thousands of badge doses from hard copy records for the period 1960–76, and literature-based dose data for the period before 1960 (Simon *et al*, 2006). This dose reconstruction, although imperfect, is likely to be superior to proxy measures of exposure such as job title. A further limitation is that offspring cancers were ascertained by parent report. Although medical record validation is preferred, the potential success was judged to be prohibitively low, given that many relevant diagnoses occurred decades ago and only the parent contact information was available. However, the accuracy of parent-reported offspring cancers is likely to be high based on the level of confirmation of diagnoses in previous analyses (e.g., 98% of haematopoietic malignancies self-reported by the technologists were confirmed in medical record review) (Linet *et al*, 2005). Moreover, the absence of an overall increase of cancer compared to rates from two different registries suggests that over-reporting was not a substantial issue with the caveat that conclusions from registry comparisons are limited by differences in geographic coverage and study period. Also, participation bias may have influenced our risk estimates. In our experience, parents of children with cancer are more likely to participate in studies than those with healthy children, thereby tending to produce a greater than expected numbers of cases. However, the overall childhood cancer rates in USRT offspring were similar to the registry rates, suggesting that participation bias did not substantially influence incidence estimates. Participation could bias risk estimates if it was related to both having a child with cancer and exposure level, although we have no data to characterize the direction or magnitude of this potential bias.

## Conclusions

Although our sample size is insufficient to detect small increases in risk, we can conclude with certainty that the risk of cancer is not greatly increased in offspring of radiologic technologists.

## Figures and Tables

**Table 1:** Characteristics of offspring of members of the USRT cohort (*n*=104 461)^a^

	**Birth year cohort of offspring**							
	**All years**	**1921–1930**	**1931–1940**	**1941–1950**	**1951–1960**	**1961–1970**	**1971–1980**	**1980–1984**
No. of offspring of female RTs	80 396 (226)	19 (0)	178 (0)	1420 (2)	9131 (36)	23 251 (77)	31 232 (79)	15 165 (32)
								
% *Male*	51 (54)	63 (0)	54 (0)	52 (50)	51 (67)	51 (49)	51 (53)	51 (53)
Mean *in utero* dose, mSV	0.2 (0.3)	0 (ND)	0.7 (ND)	0.4 (0)	0.6 (0.7)	0.1 (0.1)	0.2 (0.3)	0.2 (0.1)
Mean preconception dose, mSV	3.4 (3.4)	0 (ND)	8.1 (ND)	9.0 (0)	7.8 (5.4)	4.2 (4.1)	1.9 (2.5)	2.2 (1.5)
								
% *Female*	49 (46)	37 (0)	47 (0)	48 (50)	49 (33)	49 (51)	49 (47)	49 (47)
Mean *in utero* dose, mSV	0.2 (0.2)	0.7 (ND)	0.8 (ND)	0.4 (0)	0.6 (0.4)	0.1 (0.1)	0.2 (0.2)	0.2 (0.1)
Mean preconception dose, mSV	3.5 (2.8)	4.9 (ND)	9.5 (ND)	9.3 (18.1)	8.1 (5.2)	4.2 (2.7)	2.0 (2.1)	2.2 (2.0)
								
No. of offspring of male RTs	24 065 (68)	0 (0)	50 (0)	977 (3)	3972 (13)	6668 (23)	8828 (23)	3570 (6)
								
% *Male*	52 (60)	0 (0)	52 (0)	49 (67)	52 (62)	52 (83)	52 (43)	52 (33)
Mean preconception dose, mSV	17 (20)	—	42 (ND)	43 (0)	38 (40)	18 (24)	6.9 (3.1)	8.9 (14.2)
								
% *Female*	48 (40)	0 (0)	48 (0)	51 (33)	48 (38)	48 (17)	48 (57)	48 (67)
Mean preconception dose, mSV	17 (13)	—	51 (ND)	39 (58)	37 (38)	19 (6.9)	7.4 (4.0)	8.3 (4.3)

ND=not determined; USRT=US radiologic technologist.

Corresponding case statistics are shown in parentheses.

a 1489 subjects had missing data on gender.

**Table 2:** Age-group-specific childhood cancer rates per million person-years (*n*) reported by USRT cohort members by age group (*n*=105 940)

	**Offspring of female age group (years)**					**Offspring of male age group (years)**				
	**0–19**	**0–4**	**5–9**	**10–14**	**15–19**	**0–19**	**0–4**	**5–9**	**10–14**	**15–19**
*Haematological malignancies*	77 (111)	86 (37)	75 (30)	57 (21)	84 (25)	76 (34)	122 (15)	49 (6)	44 (5)	83 (8)
Leukaemia	33 (63)	72 (29)	52 (21)	19 (7)	20 (6)	47 (21)	97 (12)	49 (6)	0 (0)	31 (3)
Lymphoma	44 (48)	15 (6)	22 (9)	38 (14)	64 (19)	29 (13)	24 (3)	0 (0)	44 (5)	52 (5)
										
*Solid tumours*	80 (115)	116 (47)	50 (20)	52 (17)	97 (31)	76 (34)	89 (11)	25 (3)	61 (7)	134 (13)
CNS	37 (34)	25 (10)	22 (9)	25 (9)	20 (6)	36 (12)	24 (3)	8 (1)	26 (3)	52 (5)
Neuroblastoma	11 (16)	35 (14)	5 (2)	0 (0)	0 (0)	2 (1)	8 (1)	0 (0)	0 (0)	0 (0)
Kidney/Wilms' tumour	10 (14)	25 (10)	10 (4)	0 (0)	0 (0)	4 (2)	16 (2)	0 (0)	0 (0)	0 (0)
Retinoblastoma	4 (6)	15 (6)	0 (0)	0 (0)	0 (0)	11 (5)	32 (4)	8 (1)	0 (0)	0 (0)
Thyroid cancer	4 (6)	0 (0)	0 (0)	0 (0)	6 (20)	2 (1)	0 (0)	0 (0)	0 (0)	10 (1)
Other solid tumours^a^	27 (39)	17 (7)	12 (5)	27 (10)	57 (17)	29 (13)	8 (1)	8 (1)	35 (4)	72 (7)
Total childhood cancers	156 (226)	202 (82)	125 (50)	109 (38)	181 (56)	158 (68)	211 (26)	74 (9)	105 (12)	227 (21)

CNS=central nervous system; USRT=US radiologic technologist.

The numbers in parentheses are the number of cases in each age group.

a Other solid tumours included reported offspring cancers: appendix (*n*=1), bone (*n*=4), breast (*n*=1), bladder (*n*=1), colon (*n*=1), Ewing's sarcoma (*n*=4), germ-cell tumours (*n*=3), leiomyosarcoma (*n*=1), liver tumours (*n*=2), lung pulmonary blastoma (*n*=1), oral (*n*=1), ovarian (*n*=2), pancreas (*n*=1), parotid gland (*n*=2), primitive neural ectodermal tumours (*n*=2), rhabdomyosarcoma (*n*=5), soft tissue sarcoma (*n*=2), other sarcomas (*n*=8), stomach (*n*=1), teratoma (*n*=2), testicular cancer (*n*=4), and uterine (*n*=3).

**Table 3:** Cox proportional hazards regression modelling of the association between *in utero* IR exposure and childhood cancer in the offspring of female RTs (*n*=81 262)

**Cancer type**	**Person-years**	**Cases**	**HR^a^**	**95% CI**	***P*-trend**
*Leukaemia*					
0 mGy	637 994	28	1.0	ref.	
>0–0.17 mGy	369 381	17	1.1	0.6–2.0	
0.18–1.0 mGy	381 244	15	0.9	0.5–1.8	
1.01–12.6 mGy	56 252	3	1.1	0.3–3.7	0.72
					
*Lymphoma*					
0 mGy	637 994	14	1.0	ref.	
>0–0.17 mGy	369 381	16	2.3	1.1–4.9	
0.18–1.0 mGy	381 244	14	1.8	0.9–3.9	
1.01–12.6 mGy	56 252	4	2.7	0.9–8.7	0.32
					
*Solid tumours*					
0 mGy	637 994	51	1.0	ref.	
>0–0.17 mGy	369 381	30	1.0	0.6–1.6	
0.18–1.0 mGy	381 244	29	0.9	0.6–1.5	
1.01–12.6 mGy	56 252	5	1.2	0.5–3.1	0.44
					
*Childhood cancers overall*					
0 mGy	637 994	93	1.0	ref.	
>0–0.17 mGy	369 381	63	1.2	0.9–1.7	
0.18–1.0 mGy	381 244	58	1.1	0.8–1.5	
1.01–12.6 mGy	56 252	12	1.4	0.8–2.6	0.41

CI=confidence interval; HR=hazard ratio; IR=ionising radiation; RT=radiologic technologist.

a Adjusted for birth year.

**Table 4:** Standardised incidence ratios comparing childhood cancer incidence rates in the offspring of RTs to SEER and Connecticut registry rates (*n*=100 115)^a^

	**Offspring of females**						**Offspring of males**					
	**Males**			**Females**			**Males**			**Females**		
	**O**	**E**	**O/E (95% CI)**	**O**	**E**	**O/E (95% CI)**	**O**	**E**	**O/E (95% CI)**	**O**	**E**	**O/E (95% CI)**
*SEER registry comparison*												
Leukaemia	37	30	1.2 (0.9–1.7)	26	24	1.1 (0.7–1.6)	11	9	1.3 (0.6–2.2)	8	7	1.2 (0.5–2.4)
Lymphoma	33	20	1.7^b^ (1.2–2.4)	14	14	1.0 (0.6–1.7)	10	6	1.7 (0.8–3.1)	3	4	0.8 (0.2–2.2)
All sites	120	114	1.1 (0.9–1.3)	101	99	1.0 (0.8–1.2)	39	33	1.2 (0.8–1.6)	24	29	0.9 (0.5–1.3)
												
*Connecticut registry comparison*												
Leukaemia	37	32	1.2 (0.8–1.6)	26	24	1.1 (0.7–1.6)	11	9	1.2 (0.6–2.1)	8	7	1.2 (0.5–2.3)
Lymphoma	33	21	1.6^b^ (1.1–2.2)	14	14	1.0 (0.5–1.7)	10	6	1.6 (0.8–2.9)	3	4	0.7 (0.2–2.1)
All sites	120	113	1.1 (0.9–1.3)	101	99	1.0 (0.8–1.2)	41	36	1.1 (0.8–1.5)	24	28	0.9 (0.6–1.3)

E=expected; O=observed; RT=radiologic technologist; SEER=Surveillance Epidemiology and End Results.

a Excludes data on 5826 subjects with missing gender or whose parent reported a race other than white.

b *P*<0.05.
