# Supplementary material for: Childhood cancer in the offspring born in 1921–1984 to US radiologic technologists
Source: Br J Cancer. 2008 Jul 29;99(3):545–50. doi: 10.1038/sj.bjc.6604516 (PMC2527813; doi:10.1038/sj.bjc.6604516)
Supplement: Supplementary Tables 1 and 2 [file 6604516x1.doc]

**Supplementary table 1.** Characteristics of radiologic technologists who responded to the first and second questionnaire.

|  | **All participants**  **(n=70,859)** | | | |  | **Participants**  **with eligible children***  **(n=49,557)** | | | |
| --- | --- | --- | --- | --- | --- | --- | --- | --- | --- |
|  | **Female RTs**  **n=55,771 (79%)** | | **Male RTs**  **n=15,088 (21%)** | |  | **Female RTs**  **n=38,557 (78%)** | | **Male RTs**  **n=11,000 (22%)** | |
|  | **n** | **%** | **n** | **%** |  | **n** | **%** | **n** | **%** |
|  |  |  |  |  |  |  |  |  |  |
| **Birth year cohort** |  |  |  |  |  |  |  |  |  |
| 1898-1920 | 949 | 1.7 | 495 | 3.3 |  | 489 | 1.3 | 370 | 3.4 |
| 1921-1930 | 2,561 | 4.6 | 1,417 | 9.4 |  | 1,797 | 4.7 | 1,170 | 10.6 |
| 1931-1940 | 7,708 | 13.8 | 2,606 | 17.3 |  | 6,382 | 16.6 | 2,208 | 20.1 |
| 1941-1950 | 19,571 | 35.1 | 5,643 | 37.4 |  | 15,502 | 40.2 | 4,427 | 40.3 |
| 1951-1960 | 24,947 | 44.8 | 4,927 | 32.7 |  | 14,385 | 37.3 | 2,825 | 25.7 |
| 1961-1970 | 8 | 0.01 | 0 | 0 |  | 2 | 0.01 | 0 | 0 |
|  |  |  |  |  |  |  |  |  |  |
| **Decade first employed^†^** |  |  |  |  |  |  |  |  |  |
| 1923-1930 | 34 | 0.1 | 6 | 0.04 |  | 14 | 0.04 | 3 | 0.03 |
| 1931-1940 | 370 | 0.7 | 122 | 0.8 |  | 219 | 0.6 | 98 | 0.9 |
| 1941-1950 | 2312 | 4.0 | 1,096 | 7.3 |  | 1,641 | 4.4 | 915 | 8.6 |
| 1951-1960 | 8072 | 14.5 | 2,402 | 15.9 |  | 6,601 | 17.5 | 2,029 | 19.0 |
| 1961-1970 | 18814 | 33.7 | 4,446 | 29.5 |  | 14,835 | 39.3 | 3,607 | 33.8 |
| 1971-1980 | 24807 | 44.5 | 6,504 | 43.1 |  | 14,331 | 38.0 | 3,988 | 37.4 |
| 1981-1990 | 190 | 0.3 | 47 | 0.3 |  | 109 | 0.3 | 21 | 0.2 |
|  |  |  |  |  |  |  |  |  |  |
| **Number reporting children** | 44,101 | 79 | 12,240 | 81 |  |  |  |  |  |
|  |  |  |  |  |  |  |  |  |  |
| **Median number**  **of children reported** | 2 |  | 2 |  |  | 2 |  | 2 |  |
|  |  |  |  |  |  |  |  |  |  |

^*^Limited to RTs whose children were born in 1984 or earlier.

^†^Year first worked reported on the first questionnaire was used for classification; 1,637 individuals (1,172 females and 465 males) had missing information on year first worked.

**Supplementary table 2.** Cox proportional hazards regression models of the association between preconception exposure to occupational IR in the offspring of radiation technologists (n=105,940).

| **Cancer type** | **Person-years** | **cases** | **HR** | **95% CI** | | **p-trend** |
| --- | --- | --- | --- | --- | --- | --- |
| **Offspring of females (n=81,262)^*^** |  |  |  |  |  |  |
| **Leukemia** |  |  |  |  |  |  |
|  |  |  |  |  |  |  |
| <0.43 mGy | 368,856 | 16 | 1.0 | ref. | |  |
| 0.43-1.49 mGy | 351,500 | 12 | 0.8 | 0.4-1.8 | |  |
| 1.50-3.57 mGy | 353,446 | 18 | 1.3 | 0.6-2.6 | |  |
| >3.57 mGy | 371,069 | 17 | 1.1 | 0.5-2.2 | | 0.14 |
|  |  |  |  |  |  |  |
| **Lymphoma** |  |  |  |  |  |  |
|  |  |  |  |  |  |  |
| <0.43 mGy | 368,856 | 16 | 1.0 | ref. | |  |
| 0.43-1.49 mGy | 351,500 | 6 | 0.4 | 0.1-0.9 | |  |
| 1.50-3.57 mGy | 353,446 | 11 | 0.7 | 0.3-1.6 | |  |
| >3.57 mGy | 371,069 | 15 | 0.9 | 0.4-1.8 | | 0.23 |
|  |  |  |  |  |  |  |
| **Solid tumors** |  |  |  |  |  |  |
|  |  |  |  |  |  |  |
| <0.43 mGy | 368,856 | 30 | 1.0 | ref. | |  |
| 0.43-1.49 mGy | 351,500 | 27 | 0.9 | 0.5-1.6 | |  |
| 1.50-3.57 mGy | 353,446 | 28 | 1.0 | 0.6-1.6 | |  |
| >3.57 mGy | 371,069 | 30 | 1.0 | 0.6-1.7 | | 0.92 |
|  |  |  |  |  |  |  |
| **Childhood cancers overall** |  |  |  |  |  |  |
|  |  |  |  |  |  |  |
| <0.43 mGy | 368,856 | 62 | 1.0 | ref. | |  |
| 0.43-1.49 mGy | 351,500 | 45 | 0.8 | 0.5-1.1 | |  |
| 1.50-3.57 mGy | 353,446 | 57 | 1.0 | 0.7-1.4 | |  |
| >3.57 mGy | 371,069 | 62 | 1.0 | 0.7-1.4 | | 0.15 |
|  |  |  |  |  |  |  |
| **Offspring of males (n=24,678)^†^** |  |  |  |  |  |  |
| **Hematological malignancies** |  |  |  |  | |  |
| <0.67 mGy | 116,716 | 9 | 1.0 | ref. | |  |
| 0.67-4.92 mGy | 108,984 | 8 | 0.9 | 0.3-2.4 | |  |
| 4.93-15.26 mGy | 108,123 | 10 | 1.1 | 0.4-2.9 | |  |
| 15.27-81.92 mGy | 91,694 | 3 | 0.4 | 0.1-1.6 | |  |
| >81.92 mGy | 24,223 | 4 | 2.3 | 0.7-7.7 | | 0.87 |
|  |  |  |  |  | |  |
| **Solid tumors** |  |  |  |  | |  |
| <0.67 mGy | 116,716 | 7 | 1.0 | ref. | |  |
| 0.67-4.92 mGy | 108,984 | 8 | 1.7 | 0.6-4.7 | |  |
| 4.93-15.26 mGy | 108,123 | 9 | 1.4 | 0.5-4.0 | |  |
| 15.27-81.92 mGy | 91,694 | 8 | 1.2 | 0.3-6.0 | |  |
| >81.92 mGy | 24,223 | 2 | 1.4 | 0.5-4.0 | | 0.68 |
|  |  |  |  |  | |  |
| **Childhood cancers overall** |  |  |  |  | |  |
|  |  |  |  |  | |  |
| <0.67 mGy | 116,716 | 16 | 1.0 | ref. | |  |
| 0.67-4.92 mGy | 108,984 | 16 | 1.1 | 0.5-2.3 | |  |
| 4.93-15.26 mGy | 108,123 | 19 | 1.3 | 0.7-2.6 | |  |
| 15.27-81.92 mGy | 91,694 | 11 | 0.9 | 0.4-1.9 | |  |
| >81.92 mGy | 24,223 | 6 | 1.8 | 0.7-4.6 | | 0.86 |

^*^Adjusted for birth year and in utero dose category (0 mGy, >0-0.17 mGy, 0.18-1.0 mGy, 1.01-12.6 mGy).

^†^Adjusted for birth year.
